# Investigating the bearing performance of the foundation under the combined effects of flood scouring and soaking

**DOI:** 10.1038/s41598-023-50235-9

**Published:** 2023-12-20

**Authors:** Zheng Han, Haohui Ding, Hongdi Yan, Chuicheng Zeng, Changli Li, Wendu Xie, Bangjie Fu, Yange Li

**Affiliations:** 1 School of Civil Engineering, Central South University, 68 Shaoshan Road, Changsha, 410075 Hunan China; 2 Hunan Provincial Key Laboratory for Disaster Prevention and Mitigation of Rail Transit Engineering Structures, Changsha, 410075 China; 3 The Key Laboratory of Engineering Structures of Heavy Haul Railway, Ministry of Education, Changsha, 410075 China

**Keywords:** Natural hazards, Civil engineering, Computational science

## Abstract

Bearing capacity degradation of foundations under the impact of the flood is one of the major reasons responsible for the collapse and damage to the rural buildings, posing a serious threat to the local village societies. Based on a case study of a rural building foundation had been destroyed by flooding. This paper investigated the deterioration process of rural building foundations under the combined effect of dynamic scouring and static soaking caused by flooding. Using the two-dimensional shallow water equation, erosion depth was calculated for different flood velocities. Then, the bearing capacity degradation under the combined scouring-soaking effect was analyzed using the finite element method. Finally, investigating the influence of inflow direction and building group masking on the foundation's bearing capacity. The results indicate that under the combined effect, the bearing capacity of village building foundations decreases by 47.88%, with scouring slightly more impactful than soaking. Inflow angle has minimal effect on bearing performance, while the masking effect of the building group provides better protection for the foundation of rear buildings.

## Introduction

The flood is sudden, fast, and extremely destructive^1^. Among common natural disasters around the world, floods have become one of the most serious disasters in terms of frequency, breadth of impact, and the amount of damage they caused^2,3^. Recently, especially due to global warming, the high frequency of extremely heavy rainfall events in mountainous areas and the intensification of geological disaster activities have led to unusually frequent flood disasters in mountainous areas^4^. Flood disasters are increasingly becoming the major focus of flood control and disaster mitigation in mountainous areas^5,6^.

In mountainous areas of China, rural buildings face limitations in their construction due to a weak local economy, resulting in low-quality structures with poor disaster resistance^7^. Additionally, some buildings are situated on flood-prone slopes and river banks without proper site planning or geological investigation, increasing the risk of flood erosion. Existing research identifies three main failure types of rural buildings during flooding. The first type involved high-velocity floods directly impacting and damaging the building's superstructure^8^. The second type results from prolonged soaking of foundation soil and building materials, leading to the deterioration of their physical and mechanical properties^9^. The third type of failure arises from the erosion of foundation soil caused by high sand-bearing floods, resulting in settlement issues^10^. Currently, more studies focus on the first and second types of rural building failure. However, the adverse effects of the third type on foundations are critical due to the low standard characteristics of rural building foundations. Shallow buried strip foundations are common in rural areas^11^, making them vulnerable to scouring and deterioration. Understanding the variation in foundation bearing performance under the combined actions of scouring and soaking is essential for effective flood control and resistance in mountainous regions.

Current research on the bearing performance of foundations during flooding primarily focuses on two aspects: degradation of foundation soil properties due to scouring and soaking. Scholars utilize experimental observations and numerical simulations to analyze the mechanical properties of specimens under soaking action, revealing the deterioration mechanism^12–16^. The Scouring effect is also being studied, with formulas proposed for erosion depth at building corners^11^ and masonry structural^17,18^ performance under the coupled impact of flood scouring and earthquakes. However, most studies only consider single foundation soaking from static flood action and neglect the dynamic scouring and eroding effects during flooding^19^. In reality, flood impact on rural building foundations is the joint result of short-term scouring and long-term soaking effects. Erosion removes soil from the foundation's surface, exposing it, while soaking alters the foundation soil properties. The combination of these actions significantly reduces the foundation's bearing capacity.

Furthermore, Studies on mountainous village buildings mostly focus on single building impact and soaking effects^20–22^, while the masking effect between buildings during floods is often overlooked. Little research has been done on how the building group's masking effect affects foundation bearing capacity^23,24^. Additionally, buildings in different locations vary in their ability to resist floods and the energy reduction they experience, impacting their safety differently. Key questions that require further study include understanding how the dynamic scouring process during floods affects the foundation bearing performance. Additionally, understanding the influence of combined scouring-soaking actions on the bearing capacity of foundations in building groups is a key question for future study. Moreover, village buildings' orientation is determined by builders based on topographical terrain, ventilation, and lighting conditions^25–27^. Flood flows may not necessarily be perpendicular to one side of the building, necessitating consideration of how different scouring angles affect the bearing capacity of rural building groups' foundations.

In order to reduce flood damage to rural buildings, it is necessary to study the degree of deterioration of foundations by the dynamic scouring action of floods and the subsequent static immersion action. It is also necessary to explore the deterioration of foundation bearing capacity by the layout of rural buildings and the orientation of individual buildings. In order to achieve the above demand, the research logic follows three main steps. Firstly, based on the findings of previous studies^28–30^, the paper discussed the rationality of erosion foundation model that considers vertical nonlinear flow velocity distributions for flood. Secondly, the paper analyzed the foundation's bearing performance before and after flooding by considering the combined effect of dynamic scouring and static soaking. Lastly, the paper explored the influence of different scouring angles on single building foundation bearing performance and studied the change in bearing capacity reduction caused by the masking effect of building groups.

## Methodology

In this paper, the effects of flood scouring and soaking on foundation bearing performance were studied through two main processes: flood dynamic scouring to the foundation and deterioration of the foundation's bearing capacity under the combined action of flood scouring-soaking. The above processes were further decomposed into three main physical processes: the dynamic flood process, the erosion process of the foundation soil by the flood, and the deterioration process of the foundation soil due to flood soaking.

For the dynamic flood process, the two-dimensional shallow water equation was used for simulation. The erosion process of flood on the foundation soil was calculated using an erosion model based on the vertical nonlinear distribution law of the flood, using results from the flood dynamic simulation. To analyze the effect of combined scouring-soaking on the foundation's bearing capacity, ABAQUS was used with the control variable method, referencing existing research parameters for flooded-soaked foundations. The specified models and methods adopted are as follows.

### Two-dimensional shallow water equations and numerical solutions

The two-dimensional Shallow Water Equations were derived from the Navier–Stokes equation, which suitable for granular flow with a much greater length of fluid motion compared to its depth. These equations govern mass conservation and momentum conservation.

These equations are solved on a staggered grid using the semi-Lagrangian method with a central difference format, as detailed in the findings of previous studies^31^.

### The foundation scouring model based on the vertical nonlinear distribution of flow velocity

Since the flood and erodible foundation soils are assumed to be continuous and homogeneous layers in the calculation, hence the erosion of the foundation soils by the flood can be quantified by the variation of the erosion depth with time (i.e., erosion rate). According to the erosion principle^32^ based on the momentum exchange between the fluid and the eroded bed, based on the previous studies^28,31^, the erosion rate of flood can be expressed as:

1 $$E=\frac{gh{\text{ cos}}\theta [{\text{tan}}{\varphi }_{f}-(1-{B}_{d}){\text{tan}}{\varphi }_{bed}]}{{v}_{1bot}}$$

In Eq. (1), $h$ is the flood depth; $g$ is the gravitational acceleration; $\theta$ is the slope value along the flood flow direction; ${\text{tan}}{\varphi }_{f}$ is the bulk basal friction angle of the flood, proposed by the Hungr and McDougall^33^; ${\varphi }_{bed}$ is the internal friction angle of erodible bed; and ${v}_{1bot}$ is the boundary velocity; ${B}_{d}$ is the pore pressure coefficient, which characterizes the complex variation process of pore pressure in the erodible bed under the overlying load. And in previous study^31^, the complex variation law of ${B}_{d}$ is described by introducing a random function based on the comprehensive consideration of the overlying load of the erodible bed.

From Eq. (1), the erosion rate is inversely proportional to the basal flow rate of the flood, indicating that the variation of the basal flow rate greatly affects the accuracy of the erosion rate calculation. Therefore, based on this, the model for the vertical nonlinear distribution of fluid velocity^29^ is introduced in this paper, as shown in Eq. (2).

2 $$f\left(y\right)=1+a{\text{ln}} \left(\frac{y}{h}\right)$$

**Figure 1:**
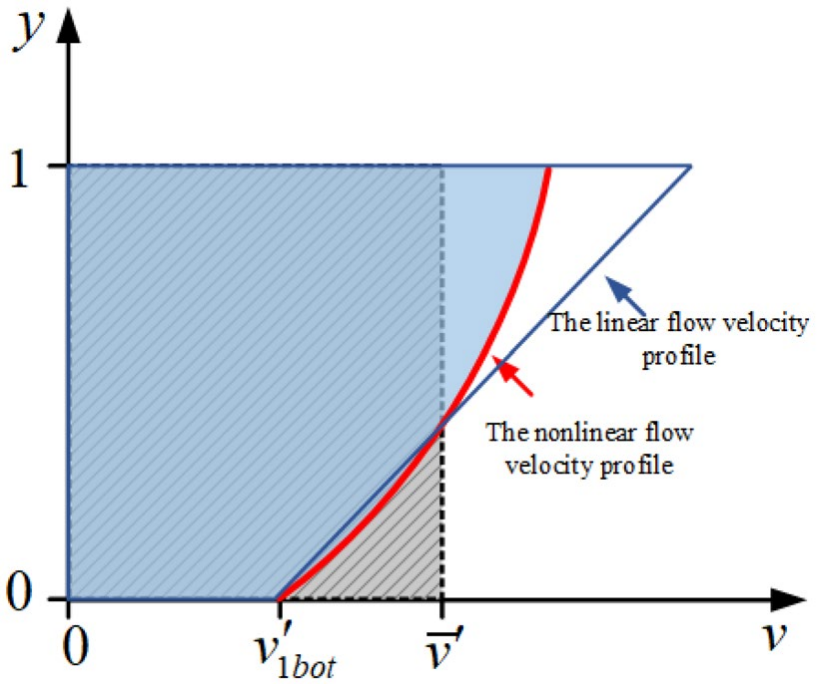
Relationship between mean and basal velocity in the vertical distribution model of flood velocity (where the y direction indicates the flow depth direction; the solid red line represents the nonlinear velocity distribution and the solid blue line represents the linear velocity profile).

In the above equation, $y$ denotes the flow depth of a point in the fluid; $a$ denotes the nonlinear distribution coefficient of flow velocity, with a suggested value of 0.30–0.35^29^. This model establishes the relationship between the mean flow velocity generated by the two-dimensional shallow water model and the basal flow velocity under the nonlinear distribution model. In this paper, the relationship between the mean flow velocity and the basal flow velocity is calculated using the Riemann integral, as shown in Fig. 1, and the basal flow velocity in the normalization model can be found by taking $y=0$ in the depth direction as follows.

3 $${v}_{1bot}{\prime}=f(y=0)$$

Finally, the relationship between the true spatial depth-averaged flow velocity and the basal flow velocity is established based on the equal ratio of the velocity in the normalized model to the true velocity distribution, and it can be written as:

4 $${v}_{1bot}=\overline{v}\times \frac{{v }_{1bot}{\prime}}{{\overline{v} }{\prime}}$$

where ${v}_{1bot}$ is the true spatial basal flow velocity.

The instantaneous erosion rate in each time step ($dt$) can be estimated by Eq. (1) when a flood depth and basal flow velocity are given, and the instantaneous erosion rate is accumulated over time to obtain the cumulative erosion depth, which can be expressed as:

5 $${d}_{sc}={\int }_{t=0}^{t}E(t)dt$$

where $E(t)$ is the instantaneous erosion rate of the foundation soil, and $t$ is the process time that flood scouring.

### Analysis of the bearing performance of foundations under the coupled effect of flood scouring-soaking on different conditions

After flood scouring, the foundation soil undergoes erosion and dragging effects, resulting in a loose and soft state due to flood soaking. These factors contribute to the change in foundation bearing capacity. Based on literature survey and field research^20,25,34,35^, it is evident that the scouring effect of floods is mainly influenced by the flood scouring angle and the masking effect of building groups. To clarify the deterioration process of foundation bearing performance under the combined effect of flood scouring-soaking, and to establish effective anti-flood reinforcement facilities, it is crucial to consider the impact of flood scouring angles and the masking effect of buildings on the foundation's bearing performance.

Before studying the deterioration mechanism of foundation bearing performance due to flood scouring, it is essential to identify the key factors affecting the change in bearing capacity. Classical theoretical analytical formulae for shallow foundations^36–38^ shows that parameters can be grouped into two categories: the mechanical properties of soil and the dimensional parameters of the foundation. This paper primarily focuses on the impact of these two aspects on foundation performance.

The ultimate bearing capacity of the foundation before and after scouring was calculated using finite element analysis with ABAQUS software. The changes in foundation bearing capacity before and after flooding were compared and analyzed. Comprehensive analysis of existing criteria and experimental research, the mechanical parameters of the soil before and after 12 h of soaking in the clay foundation soaking test by Zhao et al.^39^ Simultaneously, the infiltration depth of the flood was also introduced to fully consider the effect of soaking. The bearing capacity of the foundation under different effects was calculated by the control variable method, and then, the bearing performance of the foundation under four states, namely, no scouring and soaking, scouring only, soaking only, and scouring and soaking at the same time, was compared and analyzed. Thus, the effect of combined dynamic scouring and static soaking on the bearing performance of foundation soil was studied.

**Figure 2:**
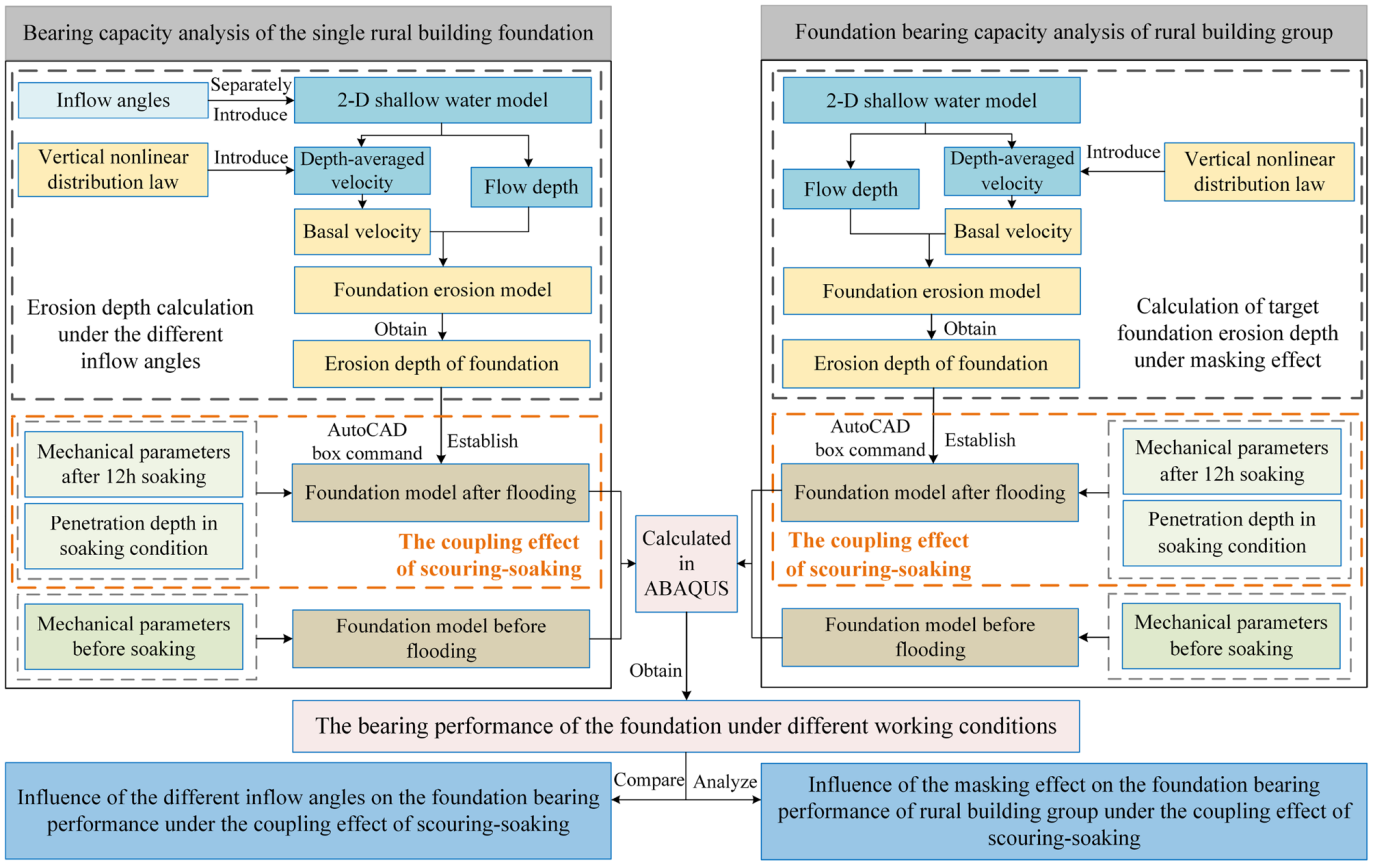
Research methodology flow chart.

To illustrate the change in bearing capacity of the target building foundation under different scouring angles and the masking effect of the building group, flood scouring simulations were conducted for the foundation models under various working conditions. Based on the obtained erosion depth, separate foundation models were established for each working condition, and the combined effect of scouring-soaking was considered to calculate the post-flooding bearing capacity of the foundation. A comparison and analysis of foundation bearing performance before and after flood scouring for different working conditions was conducted. The research process was summarized in Fig. 2. This approach allowed for the revelation of the influence of flood scouring angles and the masking effect of the building group on foundation bearing performance while accounting for the dynamic scouring and static soaking of the flood.

### Configuration of simulation

**Figure 3:**
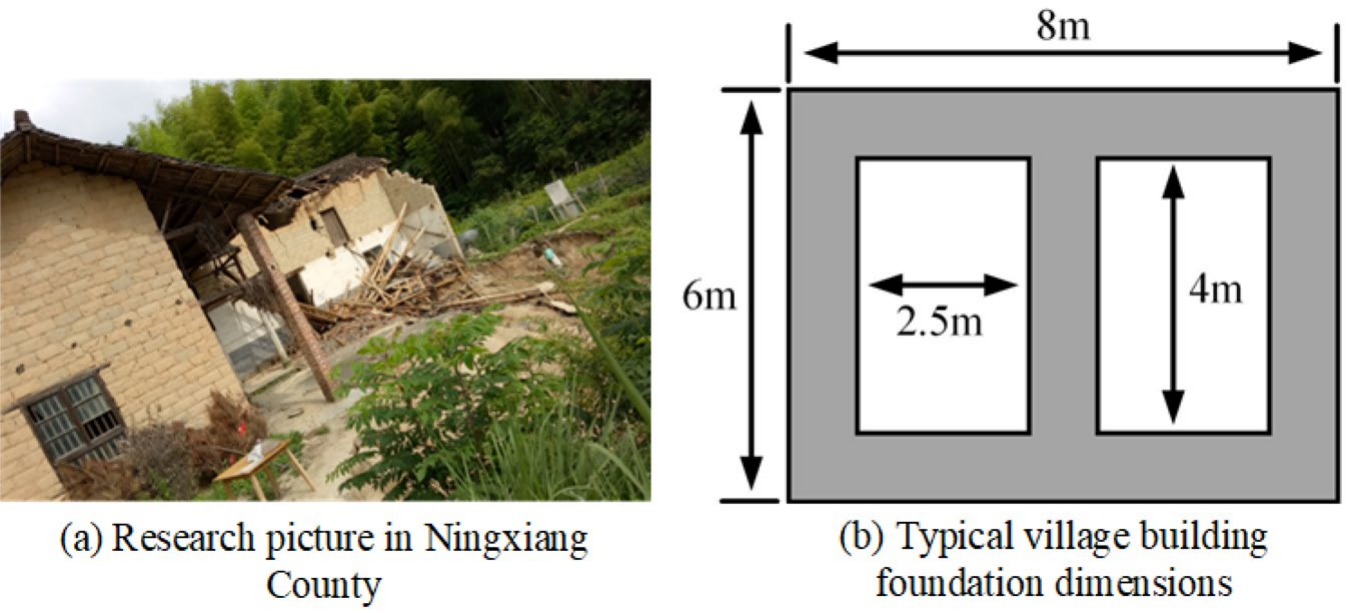
(**a**) Research pictures of village buildings damaged in the flood disaster in Ningxiang County. (**b**) Typical village building foundation dimensions (both foundation side length and burial depth are 1 m).

The masking effect of rural building groups and the scouring angle of floods are crucial factors influencing the flood scouring effect. The study aims to illustrate the variation in bearing capacity of rural building foundations before and after flood scouring, providing practical recommendations for enhancing flood resistance in rural areas. The research was conducted on a typical clay foundation, based on observations from buildings damaged during the 2017 mega-flood disaster in Ningxiang County, Hunan Province (Fig. 3a). The typical rural building form studied was a shallow-buried frame structure with two rectangular openings in the center (Fig. 3b). Using ABAQUS, a foundation model with dimensions of 24 m length, 32 m width, and 10 m height was established.

**Figure 4:**
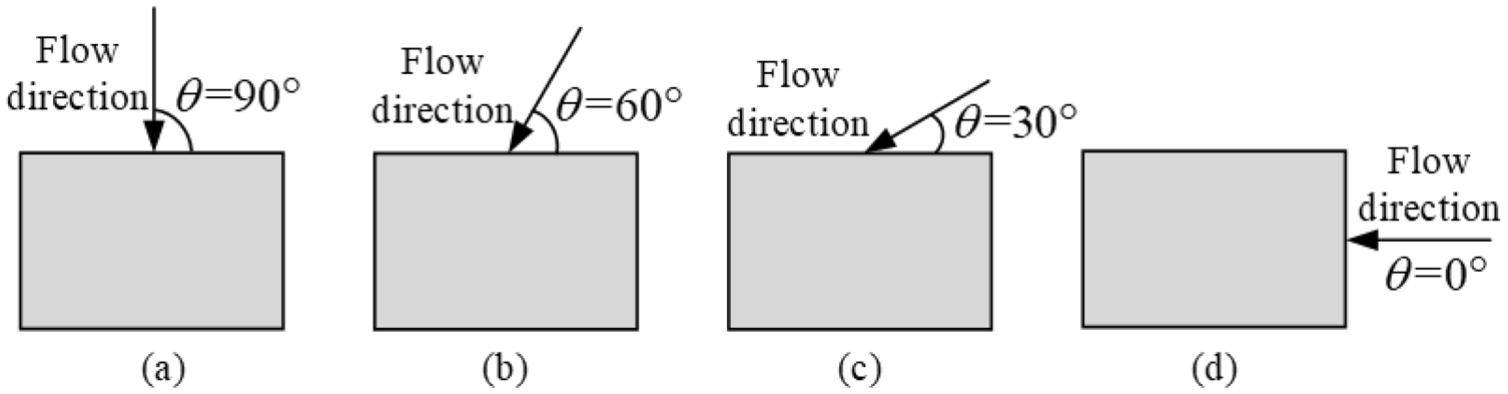
Schematic diagram of four typical scouring angles. (**a**) 90°, (**b**) 60°, (**c**) 30°, (**d**) 0°.

Buildings may have the most unfavorable spatial positions in flood^4^, that is, different scouring angles lead to varying distributions of erosion depth in the foundation soil, resulting in different reductions in foundation bearing capacity. To understand the change characteristics of foundation bearing capacity under different scouring angles, the study analyzes four different flow directions: flood direction at 90°, 60°, 30°, and 0° with respect to the long side of the building (Fig. 4).

**Figure 5:**
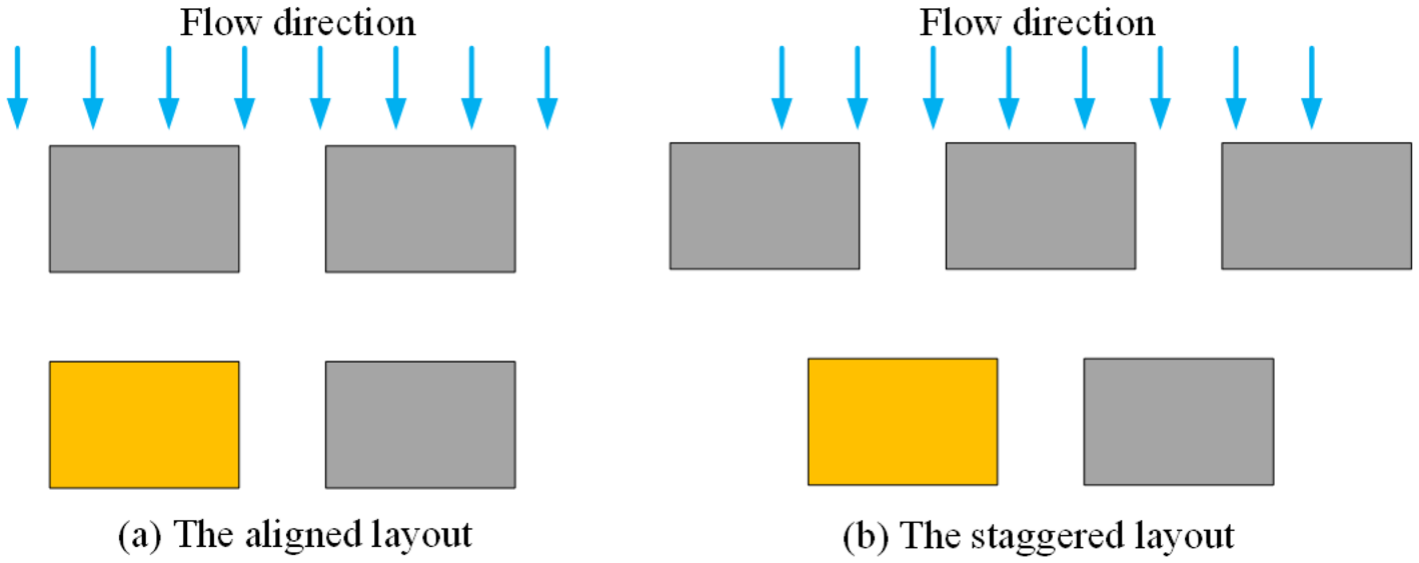
The layout of the building group masking method (blocks indicate building foundations, whereas the yellow rectangular blocks indicate the study target foundation). (**a**) aligned layout, (**b**) staggered layout.

The masking effect between building clusters is another crucial factor influencing flood scouring. Based on literature review and field research^40,41^, two main sheltering methods for rural building clusters can be identified: aligned and staggered (Fig. 5). In the simulation of the dynamic flood scouring process, the foundation soil under the building is protected from erosion, which can better highlight the scouring effect of the masking effect on the surface soil around the building. To compare the difference between these two layouts in terms of the resistance of flood scouring, this research case choosed the second row of buildings in the same position as the object of comparison analysis. This study does not consider the scattered arrangement of building rows in villages or the influence of terrain undulations when analyzing the masking effect of the building group.

**Figure 6:**
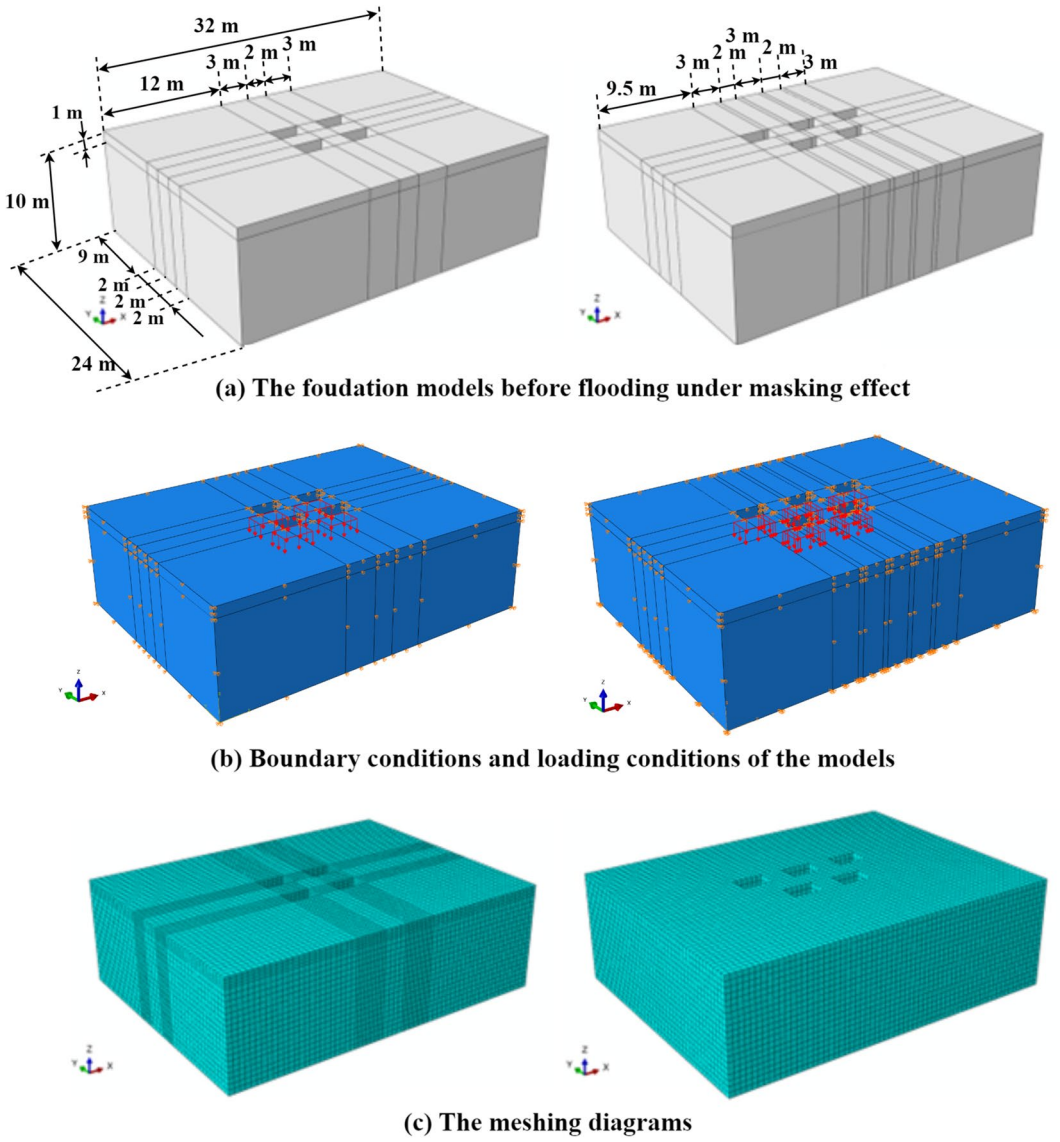
Finite element analysis of foundation before scouring under masking effect (left: aligned layout; right: staggered layout). (**a**) FEA numerical diagram. (**b**) The results of mesh division of the FEA numerical model.

To further illustrate the variation of building foundation bearing capacity before and after flooding under the masking effect, the study improved calculation efficiency by reducing the size of single building foundations and arranging them according to the proposed masking effect arrangement, as shown in Fig. 5. The selected foundation size was 2 m long and 3 m wide, with a spacing of 2 m and a burial depth of 1 m. The foundation models and mesh division results under different layout before flood scouring were shown in Fig. 6.

The boundary conditions for the model were set as follows: the foundation soil had reached equilibrium under long-term consolidation, so the displacements in the x and y directions were set to 0. For the foundation model, the displacements in the x, y, and z directions were all set to 0. At the contact surface between the foundation soil and the foundation, the displacements in the x and y directions were also set to 0.

The analysis of foundation soil bearing capacity typically involves two methods: one is the load control method, where stress values applied to the foundation soil are predetermined, and the stress and displacement fields of the foundation soil are observed. The other is the displacement control method, where the settlement values at the bottom of the foundation caused by the loads are predetermined. Due to uncertainties regarding the effect of flooding and soaking on foundation deterioration during the model calculation, and to ensure convergence in calculations while achieving a yielding state for the foundation soil, this study adopted the displacement control method with a specified 1 m displacement at the bottom of the foundation in the z-direction, with a maximum increment step of 200 to ensure that the soil could reach the yield state.

The mechanical property parameters of the soil significantly impact the foundation's bearing capacity calculation. This paper refers to the mechanical parameters provided by Zhao et al.^39^ in the laboratory model tests (as shown in Table 1) for the clay foundation before and after 12 h of flooding soaking time. For the depth of soil affected by flooding soaking, Liu et al.^42^ determined an infiltration depth of 2 m based on clay infiltration analysis in 2019. The foundation soil properties were characterized using the Mohr–Coulomb constitutive model.

**Table 1:** Table of clay parameters before and after flood soaking^39^.

State	Density (t/m^3^)	Modulus of elasticity (kPa)	Poisson’s ratio	Cohesion (kPa)	The angle of internal friction (°)
Before soaking	1.6147	300,000	0.30	32.8	20.70
Immerse for 12 h	1.9286	300,000	0.30	14.95	6.25

## Results

### Analysis of the bearing capacity of rural building foundation when considering the inflow angle

#### Analysis of the bearing capacity of the foundation of the rural building before flooding

**Figure 7:**
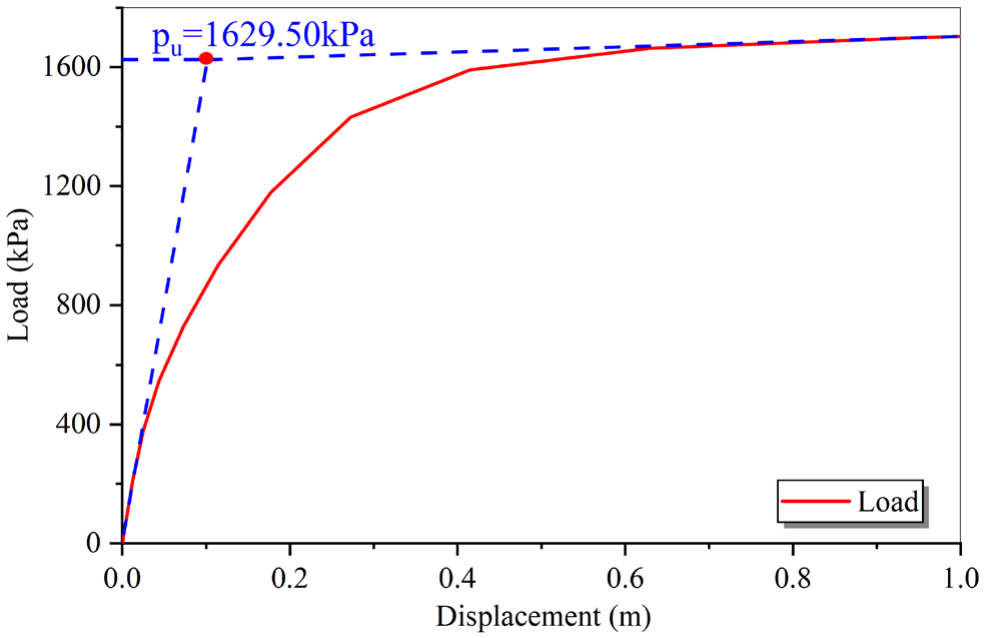
The load–displacement curve of the foundation before flooding.

In this study, the finite element software ABAQUS was used to analyze the foundation's bearing capacity before flooding. The analysis was performed using an automatic-incremental step, and the load–displacement curve was obtained (Fig. 7). The load–displacement curve became almost horizontal when the displacement reached 0.6 m, indicating that the foundation soil was close to the yield state at this point. Since the inflection point in the load–displacement curve was not evident, the tangent intersection points at the beginning and end of the curve were used to determine the ultimate bearing capacity. For the calculation model in this study, the ultimate bearing capacity of the clay foundation of the rural building before flooding was 1629.50 kPa, as detailed in Han et al.^30^.

Han et al.^30^ noted the absence of shallow foundation bearing capacity testing in their paper. To validate the finite element method's bearing capacity results, they compared them with theoretical values^43^ and measured values^44^ from similar projects in previous literature. This comparison justified the rationality of the ultimate bearing capacity results in both their paper and this study.

#### Analysis of the bearing capacity of the foundation of the rural building after flooding

In Han et al.^30^, a model based on fluid velocity distribution in a two-dimensional shallow water simulation was used to calculate foundation erosion depth. Additionally, they analyzed post-flooding foundation bearing capacity through finite element analysis, confirming the coupled model's accuracy under combined scouring-soaking conditions.

According to the simulation result of erosion depth by Han et al.^30^, it could be seen that the simulation result was consistent with the survey result^11^ and differs from the empirical calculation result by only 14.71%, which indicates that the erosion model applied in this paper was reasonable. In this study, the maximum erosion depth 0.68 m (as shown in Fig. 10a) occurs at the location of the corner point close to the direction of incoming flood flow. Detailed calculation parameters are shown in Table 2.

**Table 2:** Comparison of simulation results of erosion depth^30^.

Model parameter		Erosion depth	
Inflow angle	90°	Empirical calculation^30^	0.78 m
Scouring time	3 s	Survey results^11^	0.68 m
Mean flow velocity	12 m/s	Simulation results	0.68 m
Initial water depth	5 m		

**Figure 8:**
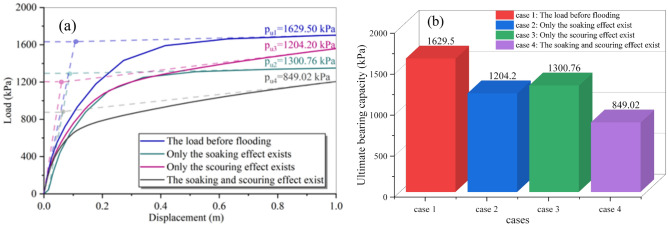
Load–displacement curves of foundation in four cases. (**a**) Bearing capacity curves, (**b**) ultimate bearing capacity.

Using the control variable method to consider flood scouring and soaking effects, the load–displacement curves of the foundation were depicted in Fig. 8. The ultimate bearing capacity decreases by 26.10% to 1204.20 kPa with only scouring action, and by 20.17% to 1300.76 kPa with only soaking, slightly higher than the bearing capacity under only scouring. When both scouring and soaking actions existed simultaneously, the ultimate bearing capacity dropped significantly to 849.02 kPa, a decrease of 47.88% compared to the pre-scouring value. The total decline of 46.27% for the superimposed effect of scouring and soaking was nearly the same as the calculated result when both actions were present, confirming the accuracy of the combined scouring-soaking model.

**Figure 9:**
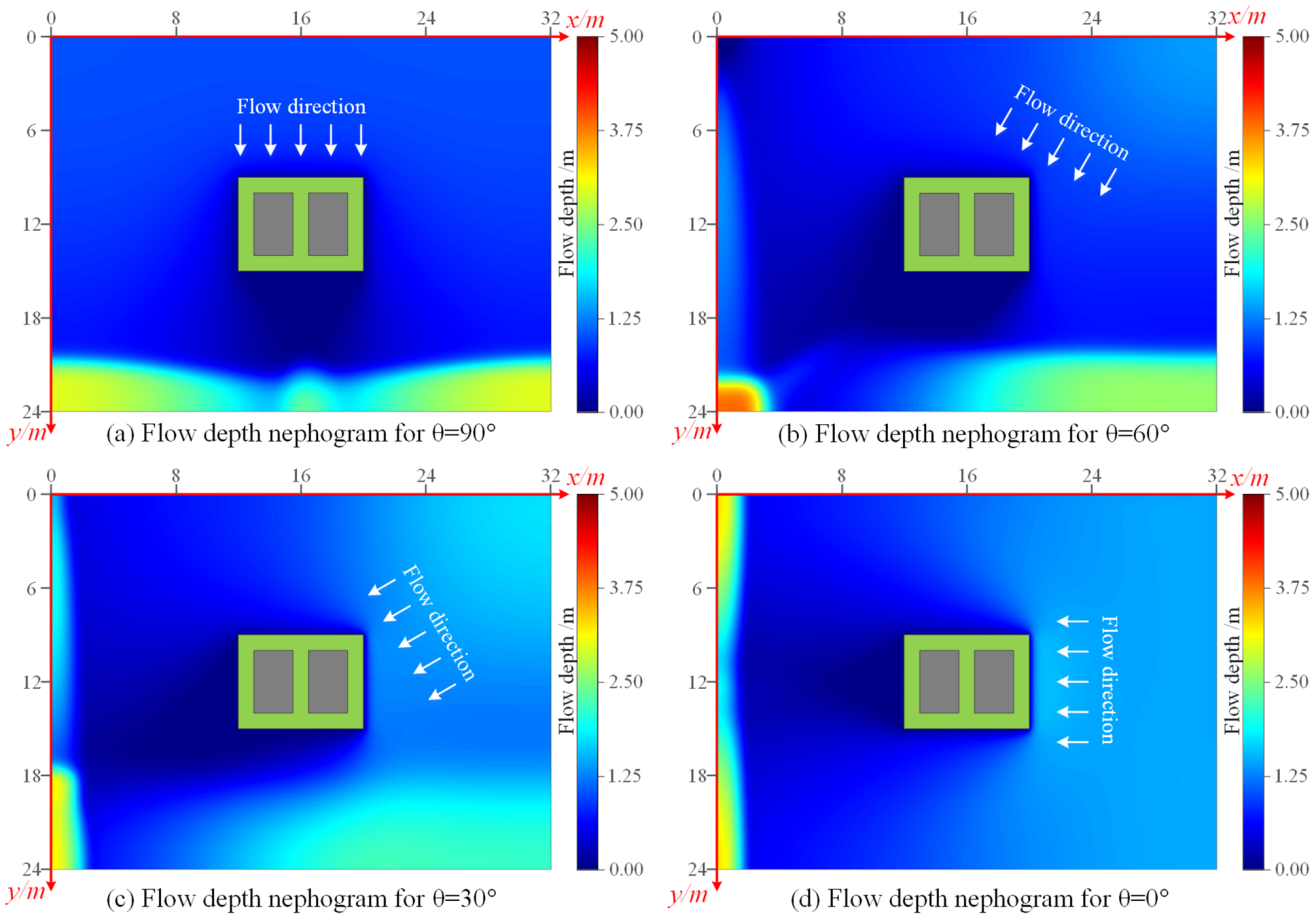
The cloud map of flow depth of flood at t = 3.00 s under four inflow angles. (**a**) 90°, (**b**) 60°, (**c**) 30°, (**d**) 0°.

**Figure 10:**
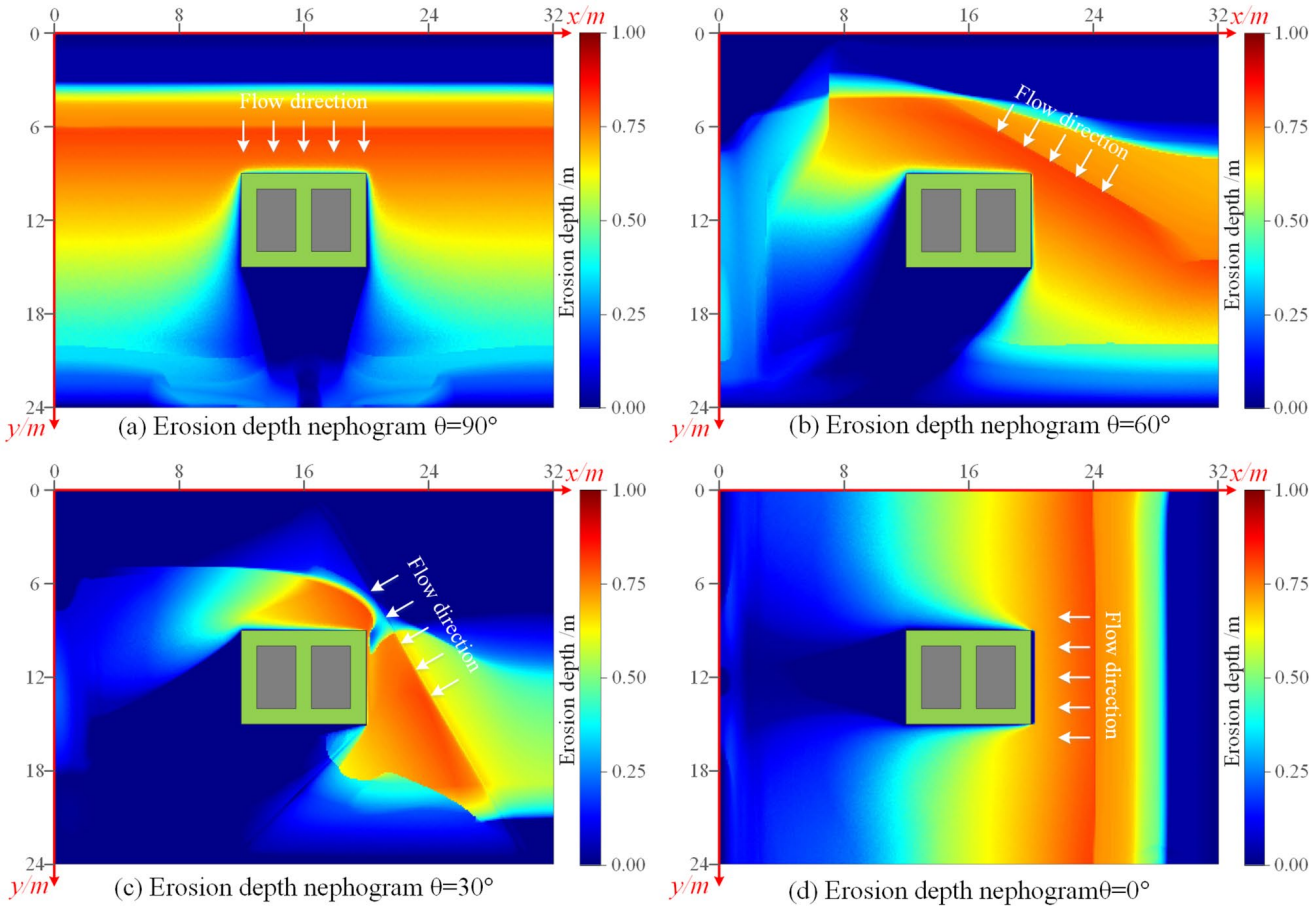
The cloud map of erosion depth at t = 3.00 s under four inflow angles. (**a**) 90°, (**b**) 60°, (**c**) 30°, (**d**) 0°.

To study the changes in the bearing capacity of the building foundation under the flooding effect of different inflow directions, based on the research by Han et al.^30^, this paper simulated the scouring of this foundation model by changing the angle of the inflow. Finally, the flow depth cloud diagrams of the flood (as shown in Fig. 9) and the simulation results of cumulative erosion depth (as shown in Fig. 10) at the last moment were obtained by the flood scouring model with different scouring angles.

When the inflow direction was perpendicular to the long side of the building (Fig. 10a), the maximum erosion depth of 0.68 m was observed at the two corner points near the inflow direction. However, when the flood flow direction was at an angle to the long side of the building (Fig. 10b–d), the erosion depth was higher on the two sides facing the flood flow direction. There was minimal erosion on the two sides of the building away from the inflow direction during the shorter simulation period. The maximum erosion depth of about 0.80 m occurred at the corner point of the building facing the flow.

**Figure 11:**
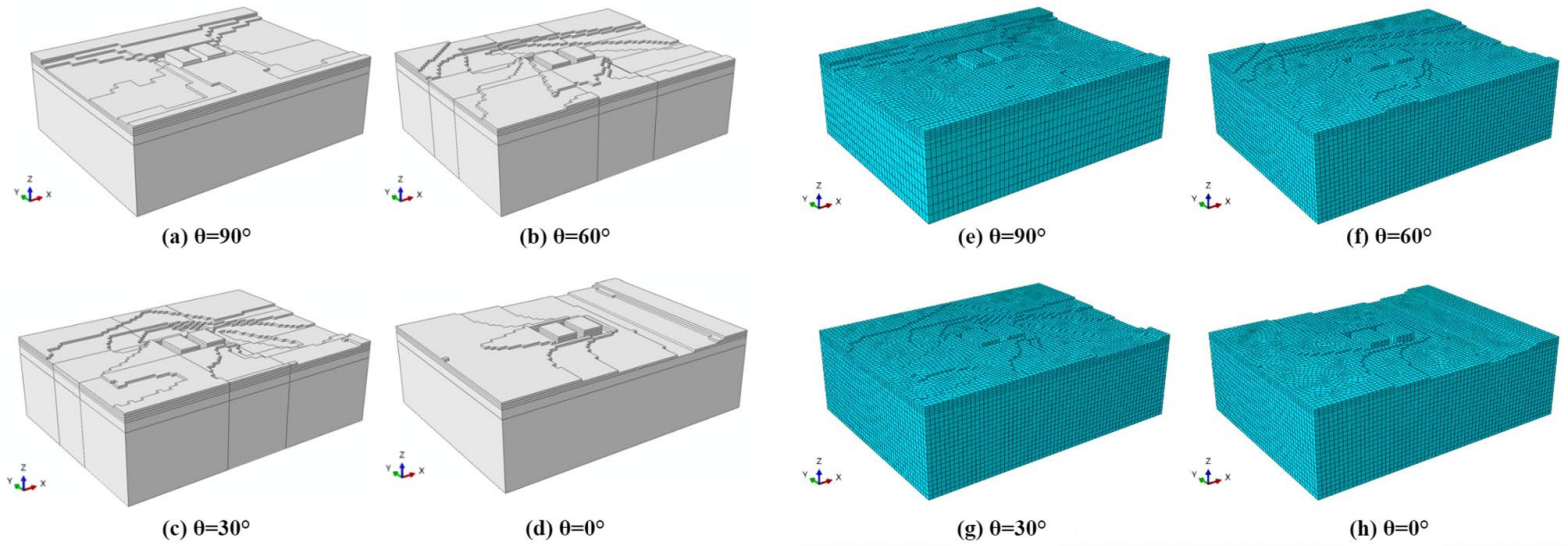
The foundation model and its meshing results after flood scour under different scour angles. (**a**,**e**) 90°, (**b**,**f**) 60°, (**c**,**g**) 30°, (**d**,**h**) 0°.

To compare the changes in the bearing capacity of the foundation before and after flooding under different inflow angles. Based on the erosion depth obtained from the erosion model, the foundation model after scouring was established (as shown in Fig. 11). The finite element method was used to analyze the bearing capacity of the foundation after flooding.

**Figure 12:**
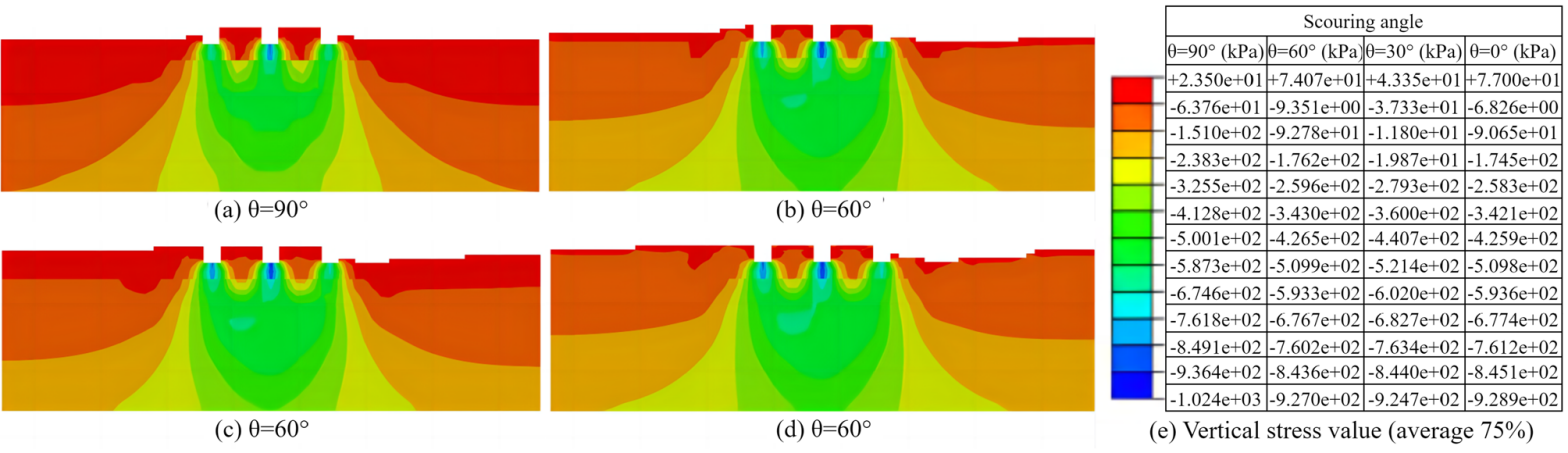
Vertical stress distributions on the specified profile after flooding at four scouring angles. (**a**) 90°, (**b**) 60°, (**c**) 30°, (**d**) 0°, (e) vertical stress value.

After equilibrium of ground stresses, the displacement loading analysis was performed on the foundation, and the vertical stress distribution on the specified profile was shown in Fig. 12. When the vertical displacement reached the maximum set value of 1 m, for the case where the inflow direction was perpendicular to the long side of the building (as shown in Fig. 12a), the maximum vertical stress of 1024.00 kPa still appeared at the center of the bottom surface of the middle strip of the foundation. For the conditions where the inflow direction is at an angle to the long side (Fig. 12b–d), the maximum vertical stress of 928.90 kPa also occurred at the center of the foundation bottom. In addition, regardless of the inflow direction, the maximum vertical stress of the foundation after flooding was smaller than that before flooding. The vertical stress in the area below the foundation surface gradually reduced from top to bottom.

**Figure 13:**
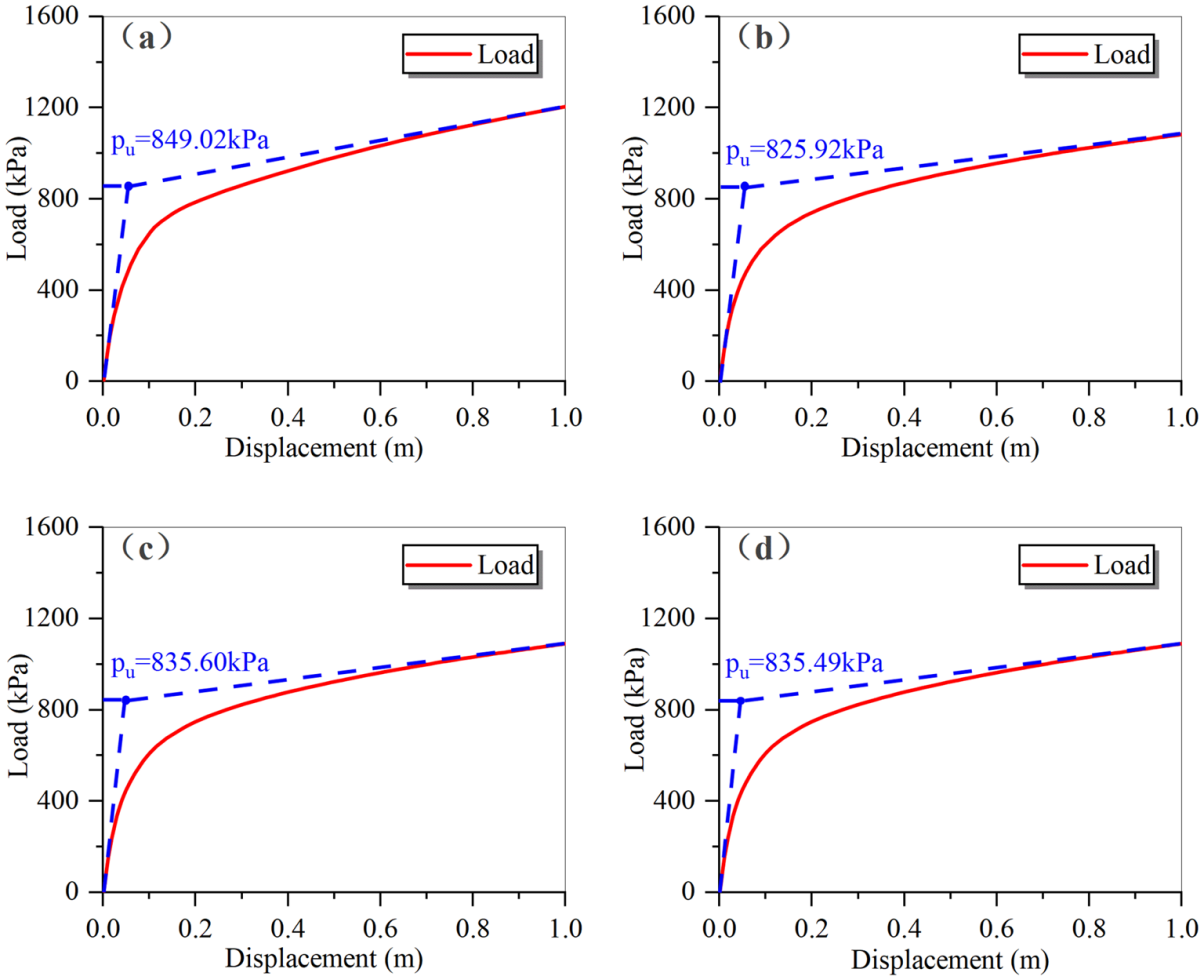
Load–displacement diagrams corresponding to foundations at four erosion angles. (**a**) 90°, (**b**) 60°, (**c**) 30°, (**d**) 0°.

Comparing the load–displacement curves under different scouring angles (Fig. 13), it could be observed that when the vertical displacement of the foundation reached 1 m, all the load–displacement curves exhibited clear inflection points, indicating that the foundation soil had reached the yield state. As previously mentioned in the section “Analysis of the bearing capacity of the foundation of the rural building before flooding”, the ultimate bearing capacity of the foundation could be determined by finding the intersection of the tangent lines at the beginning and end of the curve.

**Figure 14:**
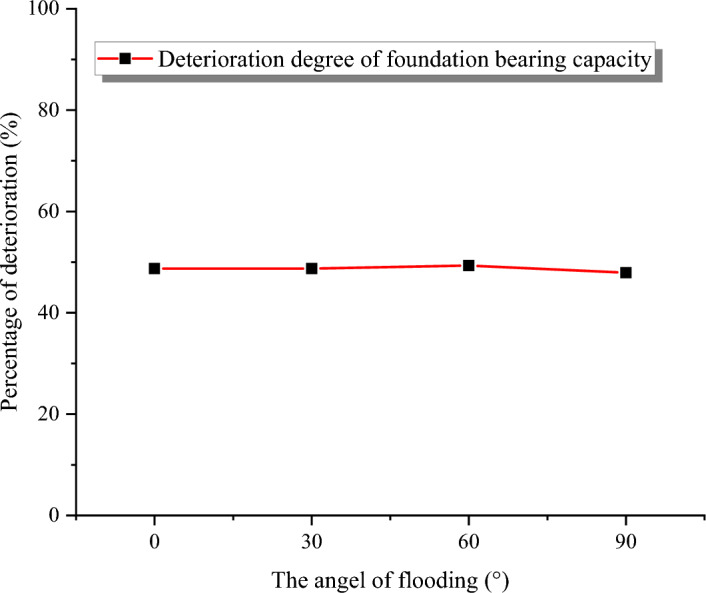
The relationship between the inflow angle and the magnitude of the decline in the bearing capacity of the foundation.

Additionally, from Fig. 13, it could be seen that the bearing capacity of the foundation under the four cases after flooding was almost unchanged, at approximately 48% of the initial bearing capacity (1629.5 kPa in Fig. 7), as shown in Fig. 14. The above data indicated that different scouring angles lead to different distributions of the erosion depth, but the influence on the bearing capacity of the foundation is minimal. In other words, the inflow angle had little impact on the bearing performance of the building foundation.

### Analysis of the bearing capacity of rural building foundation under the masking effect

#### Analysis of the bearing capacity of the foundation of the rural building before flooding

To study the changes in the foundation bearing capacity of rural buildings under the masking effect, the paper analyzed the bearing capacity of the foundations before flooding with an aligned layout and staggered layout respectively.

**Figure 15:**
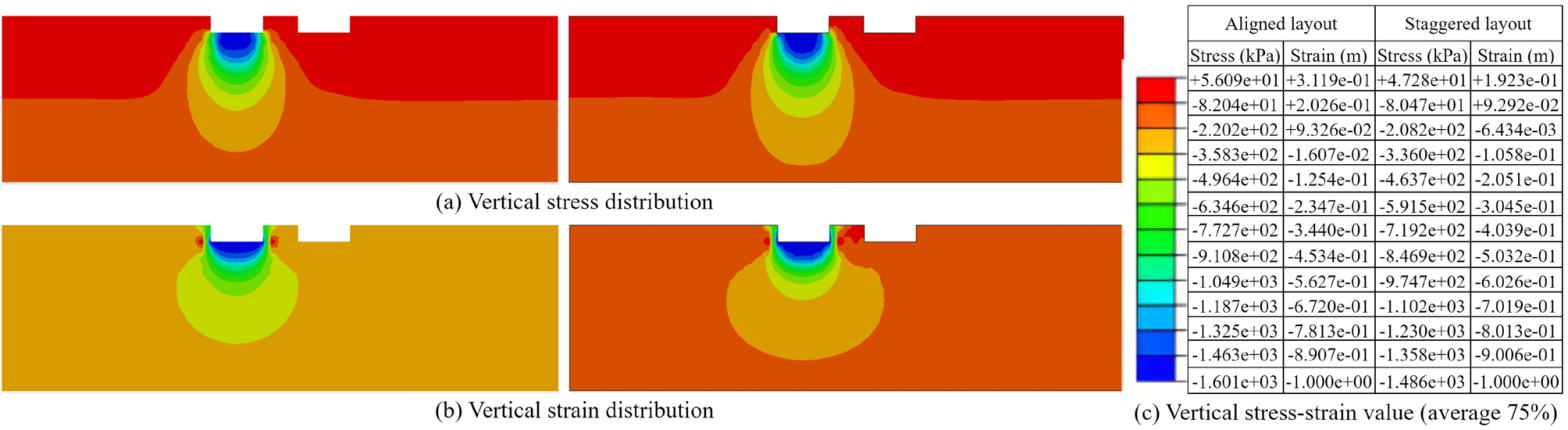
Vertical stress–strain distribution of target foundation before flooding under masking effect (Left: aligned layout; Right: staggered layout). (**a**) Vertical stress distribution. (**b**) Vertical strains distribution. (**c**) Vertical stress–strain value.

After balancing the ground stresses, the analysis of vertical stresses and vertical strains on the profile at the center of the target foundation was performed for both the aligned layout and staggered layout of the foundations (as shown in Fig. 15). From the stress distributions, it could be observed that the vertical stresses in the foundation all exhibited a significant increase. Specifically, for the aligned layout of the foundation, the maximum vertical stress reached 1601 kPa when the maximum vertical strain of the target foundation reached the set value of 1 m.

**Figure 16:**
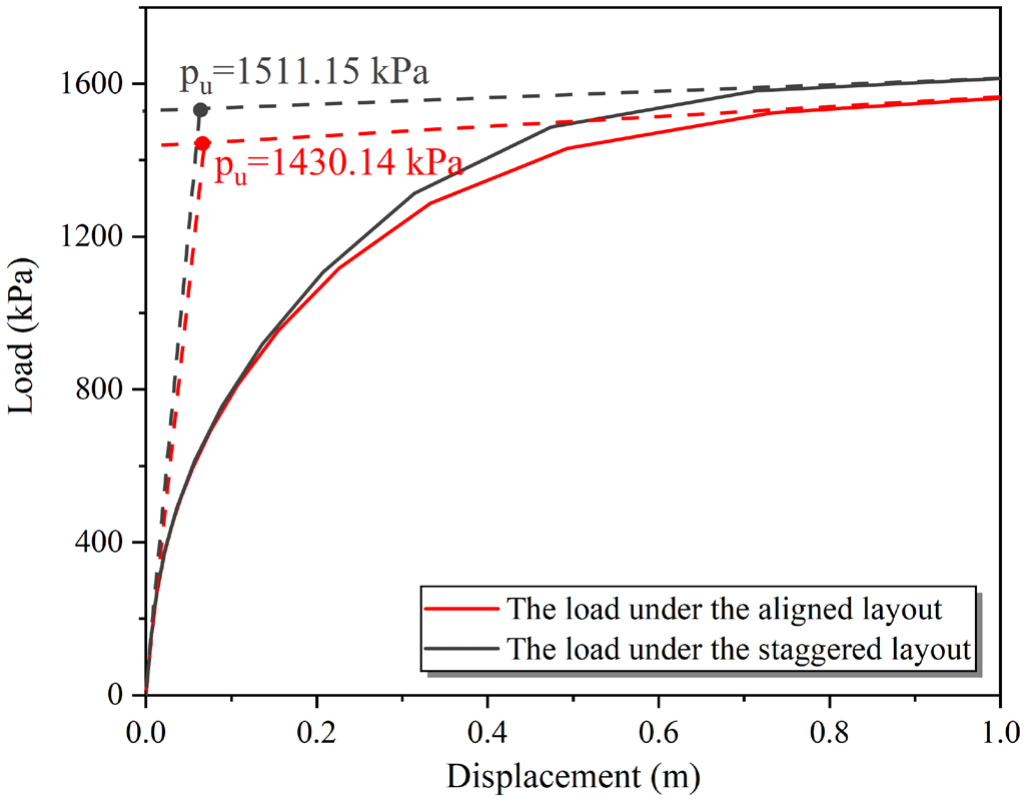
Load–displacement curves of the target building group foundation before flooding under the masking effect.

The vertical displacement and support reaction at the bottom of the foundation were analyzed based on the simulation results of vertical stress, and the load–displacement curve of the target building foundation before flooding was obtained (as shown in Fig. 16). From the load–displacement curves, it could be found that the bearing capacity of the target foundation under the aligned layout was 1430.14 kPa, and under the staggered layout was 1511.12 kPa. Based on the calculation formula of the ultimate bearing capacity of the foundation with a rectangular section proposed by Shen et al.^43^. The simulated value of the bearing capacity under the aligned layout was compared with the theoretical analysis result. The difference is only 9.28%, indicating that the ultimate bearing capacity results were reasonable. Similarly, the calculated results for the bearing capacity of the foundation under the staggered layout were also reasonable when compared with the theoretical results of Shen et al.^43^ and Li and Yang^44^. In summary, the findings of this study were in line with existing analytical solutions and measured values, confirming the reasonableness of the calculated results for the ultimate bearing capacity of the foundation under both aligned and staggered layout.

#### Analysis of the bearing capacity of the foundation of the rural building after flooding

**Figure 17:**
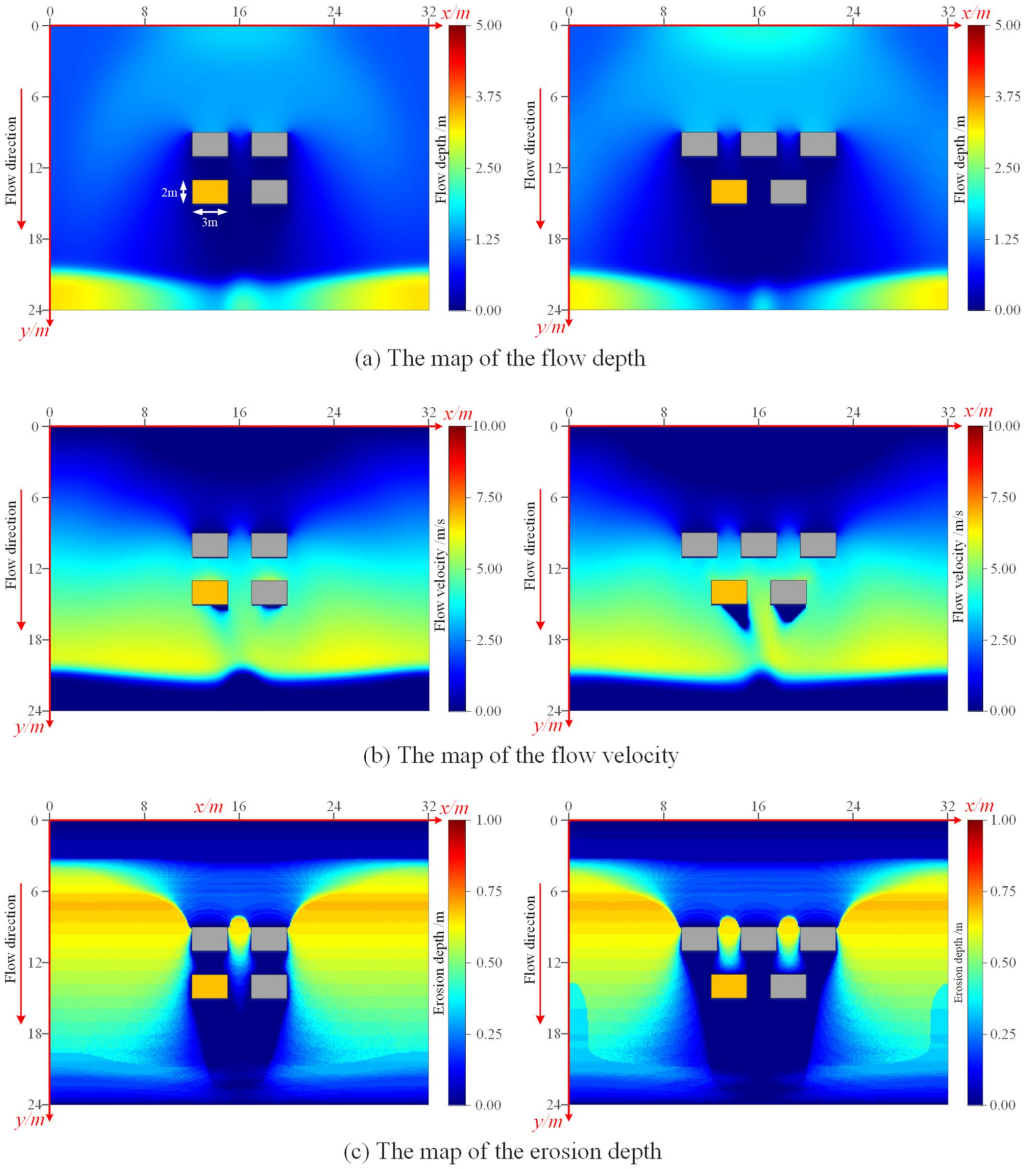
Computational cloud diagrams of the dam-break under masking effect (Left: aligned layout; Right: staggered layout). (**a**) Flow depth. (**b**) Flow velocity. (**c**) Erosion depth.

To further studied the changes in the bearing capacity of the target foundation after flooding under different spatial layouts, flood scouring simulations were performed on the foundation model with different spatial layouts without changing the parameters. The simulation results of the flow velocity, flow depth, and cumulative erosion depth at the last moment of the scouring model under both the aligned layout and staggered layout of the foundation were obtained (as shown in Fig. 17). It was observed that under the aligned layout, the distribution of flood velocity between buildings was more uniform, and the overflow capacity was stronger. Conversely, under the staggered layout, the flood in the building area overflows along the inclined direction, which aligned with the simulation result of Li^40^ for the flow velocity of the village building group.

**Figure 18:**
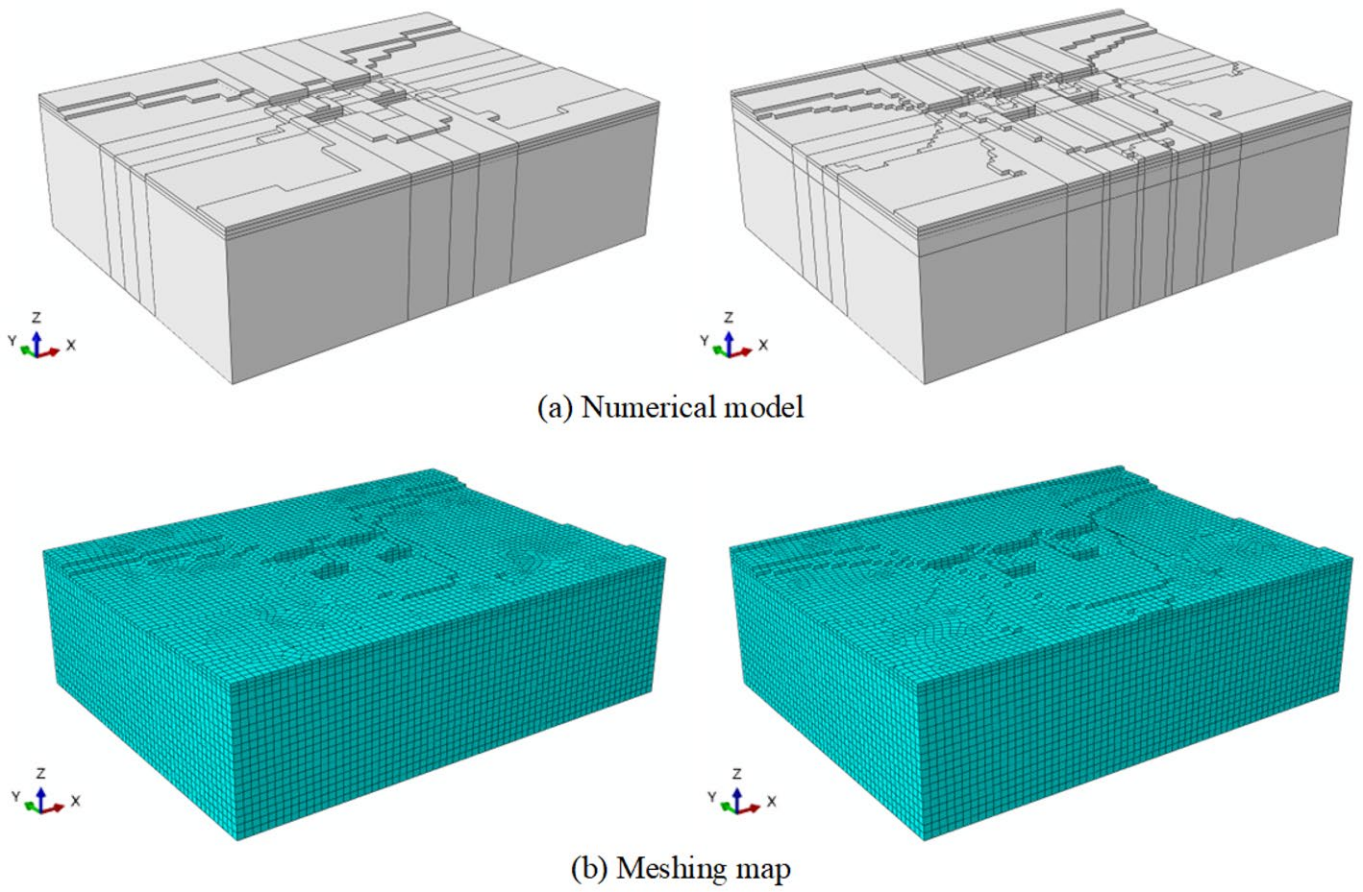
Foundation model and meshing map after erosion under masking effect (Left: aligned layout; Right: staggered layout). (**a**) Numerical model. (**b**) Meshing map.

To compare the changes in the bearing capacity of the foundation before and after flooding under different spatial distributions, this study established the foundation model after flooding according to the erosion depth obtained from the erosion model (as shown in Fig. 18). The bearing capacity of the foundation after flooding was analyzed using the finite element method. Importantly, when considering flood erosion, the changes in the mechanical properties of the soil due to the soaking effect of the flood were also taken into account, and the detailed mechanical parameters of the soil after soaking are presented in Table 1.

**Figure 19:**
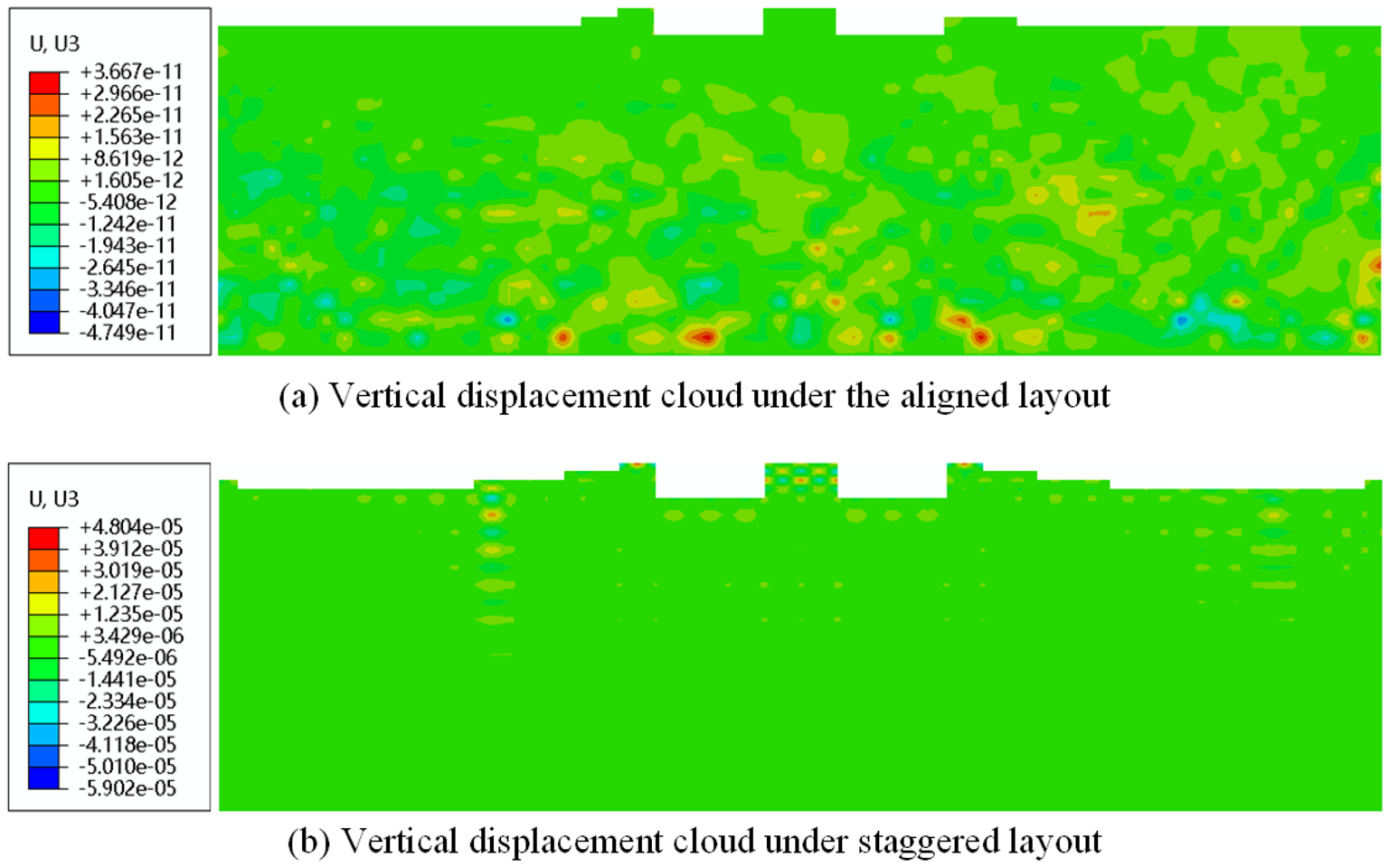
Vertical displacement clouds after flooding. (**a**) Vertical displacement cloud under aligned layout. (**b**) Vertical displacement cloud under the staggered layout.

Since the shape of the foundation soil was irregular after flooding, the balance of the ground stress was analyzed using the result import method. Meanwhile, the vertical displacement clouds after the ground stress was balanced under the masking effect were shown in Fig. 19. And the maximum vertical displacement was less than 10^−5^, which indicated that the effect of the ground stress balance was better.

**Figure 20:**
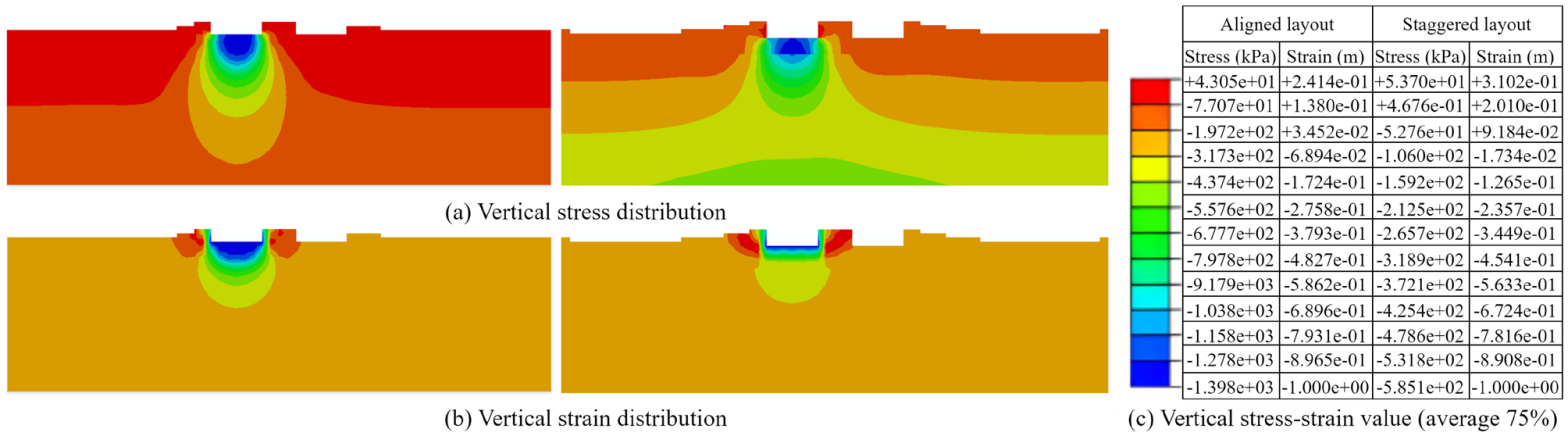
Vertical stress–strain distribution clouds of the target foundation after flooding under the masking effect (Left: aligned layout; Right: staggered layout). (**a**) Vertical stress distribution. (b) Vertical strain distribution. (**c**) Vertical stress–strain value.

After balancing the ground stress, the vertical stress and vertical strain distributions on the foundation profile were shown in Fig. 20 for both the aligned and staggered layouts. It could be observed that the maximum vertical stress still occurred in the bottom region of the foundation when the foundation reached yielding. Under the aligned layout, the maximum vertical stress was 1398.00 kPa, and under the staggered layout, the maximum vertical stress was 585.1 kPa. Both values were smaller than the maximum vertical stress before flooding.

**Figure 21:**
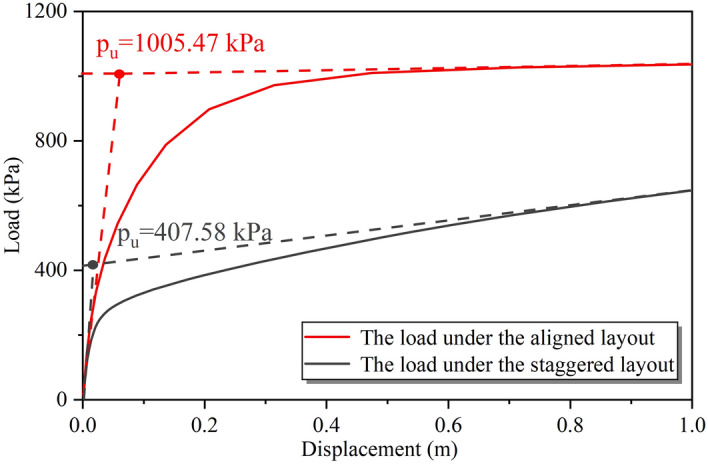
The load–displacement curves of the target building foundation after flooding under the masking effect.

The load–displacement curves of the target building foundation after flooding under different spatial distributions were shown in Fig. 21, based on the vertical stress simulation results. The ultimate bearing capacity of the foundation after flooding under the aligned layout was 1005.47 kPa, while the ultimate bearing capacity under the staggered layout was 407.58 kPa. These values represented a decrease of 29.69% and 73.03%, respectively, compared to the bearing capacity of the foundation before flooding (as shown in Fig. 16). Additionally, according to the previous calculation results^30^, the bearing capacity of a single building foundation after flooding decreased by 47.88% compared to that before flooding. This indicated that the aligned layout could significantly reduce the impact of the flood on the bearing capacity of the foundation, while the staggered layout could greatly increase the effect of the flood on the bearing performance of the foundation.

## Conclusion

To clarify the variation of the bearing performance of the building foundations under the combined effect of scouring and soaking, this study takes the foundations of rural buildings as the study case, and the conclusions are as follows.

The erosion model based on vertical nonlinear flow velocity distribution is found to be reasonable, with a difference of only 14.71% compared to empirical calculations.

When considering only erosion, the single building foundation's bearing capacity decreases by 26.10%, while for soaking, it decrease by 20.17%, slightly more than erosion alone. The combined scouring-soaking effect results in a 47.88% decrease in bearing capacity, close to the cumulative effect of separate erosion and soakage, 46.27%.

The erosion depth of the foundation has different distribution rules when the scouring angle of the flood is different. However, simulation results for bearing capacity under the combined scouring-soaking effect show values of 849.02 kPa, 825.92 kPa, 835.60 kPa, and 835.49 kPa for flood scour angles θ = 90°, θ = 60°, θ = 30°, and θ = 0°, respectively. These values represent approximately a 48% decrease compared to the pre-flood scour foundation bearing capacity of 1629.5 kPa.

For the target group buildings’ foundation, the aligned layout reduced the impact of flooding by 29.67%, while the staggered layout increases the reduction to 73.03%. These findings indicate that the aligned layout is more effective in mitigating flooding's influence on building foundation bearing performance in the back row.

## Data Availability

The datasets generated and analyzed during the current study are not publicly available because the study involves a project funded by the Chinese government, which is subject to confidentiality agreements. But these data are available from the corresponding author on reasonable request.
